# Effect of antibiotic administration on Blastocystis persistence and gut microbiome–metabolome dynamics in an irritable bowel syndrome longitudinal case study

**DOI:** 10.1099/acmi.0.000926.v4

**Published:** 2025-06-26

**Authors:** Jamie M. Newton, William J.S. Edwards, Gary S. Thompson, Eleni Gentekaki, Anastasios D. Tsaousis

**Affiliations:** 1 Laboratory of Molecular and Evolutionary Parasitology, RAPID group, School of Biosciences, University of Kent, Canterbury, UK; 2 NMR Facility, School of Biosciences, University of Kent, Canterbury, CT2 7NJ, UK; 3 School of Biosciences, University of Kent, Canterbury, CT2 7NJ, UK; 4 University of Nicosia School of Veterinary Medicine, 2414, Nicosia, Cyprus

**Keywords:** antibiotics, *Blastocystis*, gut metabolome, gut microbiome, irritable bowel syndrome (IBS)

## Abstract

**Background.** *Blastocystis*, the most prevalent microbial eukaryote in humans, has a global distribution. Studies have linked its presence with distinct gut microbiome and metabolome profiles compared to those where the organism is absent. However, the interplay of antibiotic administration, *Blastocystis* and the surrounding gut microbiome remains understudied. This case study aimed to explore antibiotic consumption and the presence of *Blastocystis* with subsequent changes in the gut microbiome and metabolome of an individual diagnosed with irritable bowel syndrome (IBS).

**Methods.** Stool samples from an IBS patient, collected at 12 time points, were tested for the presence of *Blastocystis* using real-time PCR targeting the *SSU*rRNA gene, followed by sequencing of positive samples. Illumina sequencing determined the gut microbiome composition, while one-dimensional proton NMR spectroscopy was used to analyse the metabolome composition. Statistical analyses were conducted to identify relationships between antibiotic consumption, bacterial diversity, metabolome composition and *Blastocystis* presence.

**Results.** Antibiotics significantly impacted the gut microbiome, with diversity declining early in the antibiotic course, then recovering later and post-course. *Blastocystis* was detected early, late and post-course but was not detectable mid-course, coinciding with the decline in bacterial diversity. Significant differences were observed between *Blastocystis*-positive and *Blastocystis*-negative samples, with bacterial composition significantly changing between samples collected before, early and after the antibiotic course compared to those collected mid-course. Metabolite groups, including short-chain fatty acids, amino acids and succinate, exhibited changes throughout the antibiotic course, indicating that gut metabolite composition is affected by antibiotic consumption.

**Discussion/Conclusion.** While antibiotics did not significantly impact *Blastocystis* colonization, they did cause a mid-course decline in microbial diversity and *Blastocystis* presence. The study also revealed significant alterations in important metabolites such as short-chain fatty acids and amino acids throughout the antibiotic course, with an altered metabolome observed post-course. This case study underscores the complex interactions between antibiotics, gut microbiota and metabolites, highlighting the resilience of *Blastocystis* in the gut ecosystem.

## Data Summary

Sequencing data can be found on the NIH GenBank sequence read archive under the study SRP520965. The following accession numbers correspond to samples used in the study: SRR29890273 – D0, SRR29890272 – D2, SRR29890269 – D3, SRR29890268 – D4, SRR29890267 - D5, SRR29890266 – D6, SRR29890265 - D7, SRR29890264 - D8, SRR29890263 – D10. SRR29890262 - D15, SRR29890271 - D30, SRR29890270 - D3M; https://dataview.ncbi.nlm.nih.gov/object/PRJNA1137868?reviewer=pt9tgfhstgcm68viduimd1r9gm.

## Introduction

The gut microbiome and metabolome are crucial determinants of gastrointestinal (GI) health, and their interactions can be better understood by studying them in tandem [1]. This dual approach is particularly important when investigating GI health differences across various cohorts or when external factors influence the GI tract. For example, a previous metabolomics study using ^1^H NMR spectroscopy revealed significant differences in stool metabolite composition between individuals with diarrhoea and healthy controls and between *Blastocystis* carriers and non-carriers [2]. Understanding these interactions can provide deeper insights into the complex dynamics of gut health and disease.

*Blastocystis* is a eukaryotic microbe that resides in the GI tract and has a global distribution in a broad range of animal hosts [3,4]. Epidemiological studies and phylogenetic analysis of the small subunit rRNA (*SSU* rRNA) gene have revealed over 44 different subtypes, 12 of which – subtypes 1–9, 12, 16 and 23 – have been identified in human stool samples [5,9]. *Blastocystis* was initially designated as a parasite and linked with irritable bowel syndrome (IBS) and other GI disorders [10,11]; however, more recent studies have indicated a negative correlation between the presence of the organism and GI symptoms [8,9, 12, 13], muddling its association with disease. *Blastocystis*’ genetic diversity further complicates interpretations, with many studies showing no relationship between inter- and intra-subtype diversity and disease [9,12].

Nonetheless, specific microbial profiles have been associated with the organism. For instance, *Blastocystis* presence is more common in the *Ruminococcaceae* and *Prevotella* enterotypes, rather than *Bacteroides*, and is associated with higher richness and diversity, which can be an indicator of good GI health [8,14, 15]. At the subtype level, *Blastocystis* ST3 and ST4 have been shown to have an inverse relationship with *Akkermansia* abundance, an indicator of GI health [8]. Whether *Blastocystis* is a gut ecosystem engineer, a simple colonizer or just a passenger is still unknown.

Individuals with IBS have been known to have distinct gut bacterial compositions/profiles compared to their non-IBS counterparts, making IBS treatment with antibiotics a potential influencing factor [16,19]. *In vivo* and *in vitro* studies have indicated that the gut microbiomes of individuals colonized with *Blastocystis* show a decline in abundance of genera such as *Bifidobacterium* and *Lactobacillus* [15,20, 21]. *Bifidobacterium* has a role in immunomodulation and protection of the GI epithelial cells [20,22, 23]. Therefore, the results of these studies could link *Blastocystis* to dysbiosis-induced GI symptoms and possibly IBS.

Antibiotic administration has been an effective treatment for some IBS cases and other GI conditions, while metronidazole, ciprofloxacin and rifaximin have been effective at decreasing the severity of symptoms in many clinical trials [18,19, 24,26]. However, as our understanding of the gut microbiome’s role in GI health deepens, the impact of antibiotics on gut microbiota modulation is receiving closer scrutiny. Several studies have detected significant changes in the gut microbiome composition during antibiotic treatment and, occasionally, microbiome recovery after treatment [27,29].

In this case study, we monitored the metabolome and the bacterial gut microbiome composition of a *Blastocystis*-positive IBS patient during a 14-day course of antibiotics. We also analysed *Blastocystis* presence over this 14-day period and how it is impacted by antibiotic treatment. The subject’s gut microbiome and metabolome composition were also analysed following the termination of the antibiotic course to monitor microbial diversity recovery after *Blastocystis* detection.

## Methods

### Case presentation: participant recruitment and sample collection

The study subject, previously diagnosed with IBS, presented to a hospital in Kent County (South East England) with GI symptoms and was put on a 14-day course of antibiotics. These included 500 mg amoxicillin (a third-generation penicillin antibiotic) ×2 and 500 mg clarithromycin (a second-generation macrolide antibiotic). Daily doses of 30 mg lansoprazole (proton pump inhibitor) ×2 were also prescribed. The subject was in their 40 s (41 to 45) with a Body Mass Index of 29 and a mixed diet. The subject was provided with faeces catchers (Zymo Research Cat. No. R1101-1-10) and two types of collection tubes, one containing 5 ml DNA/RNA shield (Zymo Research Cat. No. R1100-250) and the other containing 5 ml of 50% methanol. Each faecal sample was distributed in the two tubes. Faecal samples were collected just before the commencement of the treatment course, then on D2, then once daily for the remainder of the first week and then once on D8, D10 and D15 (the day after the completion of the course) for the following week. Follow-up samples were then collected at D30 and D3M (3 months after course start). The samples were stored in their respective tubes in DNA/RNA shield or methanol at −80 °C.

### DNA extraction

Two hundred milligrammes of solid stool stored in DNA/RNA shield or 200 µl liquid stool were added to 200 µl PBS (pH 7.4 RNAase free). The samples were then centrifuged for 10 min at 10,000 ***g*** at room temperature (RT). The pellet was then resuspended in the supernatant, and the DNA was extracted using the QIAamp PowerFecal Pro DNA Kit (QIAGEN; Cat. No. 51804) following the manufacturer’s protocol, and 50 µl of DNA was eluted.

#### *Blastocystis* detection

For *Blastocystis* detection, a 350 bp region of the *SSU* rRNA gene was targeted using a reaction mixture of 2 µl DNA, 500 nM of primer set PPF1 (fwd) (5′-AGTAGTCATACGCTCGTCTCAAA-3′) and R2PP (rvs) (5′-TCTTCGTTACCCGTTACTGC-3′) and 5 µl SYBR green making a full reaction volume of 10 µl. The quantitative PCR (qPCR) was run on a QuantStudio-3 real-time PCR machine with the following programme: initial denaturation 95 °C for 5 min, then 45 cycles of initial denaturation 95 °C for 5 s, annealing 68 °C for 10 s, extension 72 °C 10 s then a final extension of 72 °C for 15 s.

### Sequencing and subtype annotation

Bi-directional Sanger sequencing using the set of primers for the qPCR reaction was outsourced to and performed by Eurofins (UK). The forward and reverse nucleotide sequences were then assessed and trimmed using SnapGene Viewer Version 6.2.2 (https://www.snapgene.com/snapgene-viewer). The final trimmed consensus sequences were then used as queries to check for contamination using the basic local alignment search tool (blast) from the National Center for Biotechnology Information (https://blast.ncbi.nlm.nih.gov/Blast.cgi). Once the identity of the sequence was confirmed as *Blastocystis*, the subtype was assigned using the curated database pubMLST (https://pubmlst.org/organisms/blastocystis-spp).

### 16S rRNA gene amplicon sequencing

Novogene outsourced the high-throughput amplicon sequencing. The protocol used was based on Caporaso *et al*. [30] with some modifications. One nanogramme of DNA from extracts was used, fragmented and then adapted for paired-end sequencing. The DNA was amplified using the primer pair 515F GTGCCAGCMGCCGCGGTAA and 907R CCGTCAATTCCTTTGAGTTT, which amplifies the hypervariable region and then sequenced on the Illumina NovaSeq platform.

The raw reads were classified using the Lotus2 software [31]. The parameters and tools used are as follows: chimaera checking/removal was performed using Minimap2 [32], and Minimap2 was also used to look for off-target hits containing human DNA ‘contaminated’ reads by performing a blast search of reads against Genome Reference Consortium Human Build 38 .p14. V3–V4 region trimmed reads were then clustered into ASVs (≤ 1 nucleotide dissimilarity) using the Divisive Amplicon Denoising Algorithm 2 [33]. ASVs were taxonomically classified (to species level) using blast against the GreenGenes2 (GG2) database [34]. GG2 was chosen for its reliability (GG2 is a unified database suitable for whole-genome sequencing data and 16S data), as well as replicable results.

### Statistical analysis

Statistical analysis and data visualization were done using the RStudio 4.2.3 package; figures were primarily produced using ggplot2 [35]. Data were first rarefied to factor in changes in sequencing depth. Relative abundances of each genus were calculated in each sample, and a heatmap was constructed. Diversity index values were calculated using the Phyloseq package. Shannon, Chao1, Simpson and observed taxa values were used. These four values were analysed for statistical differences occurring between the *Blastocystis-*positive and *Blastocystis-*negative samples, as well as differences in diversity score between the ‘antibiotic positive’ time points (days 4–15) and the ‘antibiotic negative’ time points (day 0, 30 days post-antibiotics and 3 months post-antibiotics). First, a Shapiro test was used to determine the data distribution to analyse the statistical differences between the sample groups. Normally distributed data were analysed with the ANOVA test followed by the Tukey HSD test for pairwise comparison. For samples with a non-normal distribution, the Kruskal–Wallis test was used, followed up by the Dunn test (Bonferroni *P*-adjust) for pairwise comparisons. The raw diversity index values were also plotted over time. To visualize microbiome composition, compositional plots showing all taxa making up >1% of the total read counts were produced using the Microbiome package. To look for the presence of ‘bio-markers’ in the *Blastocystis-*positive samples, linear discriminant effect size (LEfSe) analysis was done [36]. LEfSe uses a combination of statistical tests to identify taxa whose high/low abundance or the presence/absence allows for the best linear discrimination/explanation of the differences observed (changes in taxa) between the two groups of samples (*Blastocystis* +ve/-ve). Principal coordinate analysis (PCoA) was also used to determine differences between the *Blastocystis* +ve/-ve groups based on overall taxa presence and distribution. Samples were plotted based on their Bray–Curtis dissimilarity matrix values. Statistical analysis was then done using Permutational Analysis of Variance (PERMANOVA) [34] to determine if the ‘centrons’ of each group (*Blastocystis* +ve/-ve) differed significantly in location. Metabolome data were visualized using the RStudio 4.2.3 package [37]. The same process was repeated for the metabolite data using Euclidean distances (principal component analysis) and PERMANOVA. A volcano plot was constructed using the log-fold changes of metabolites, and those with a significant change were annotated. The volcano plot was made using the Metaboanalyst software [38]. Lastly, statistically significant, linear discriminant taxa (as confirmed by LEfSe) were subjected to a Spearman’s rank correlation with metabolite data to show interactions between the taxa and the metabolome.

### Metabolite extraction

Two hundred milligramme solid stool stored in methanol or 200 µl liquid stool was resuspended in 4 ml methanol, and then 200 mg glass beads were added and vortexed for 30 s. The samples were then incubated at RT for 3 min and then vortexed for a further 30 s. The supernatants were then divided into 4×1 m aliquots, centrifuged at 10,000 ***g*** at 4 °C for 20 min and then lyophilized. The lyophilized desiccates were then resolubilized in 375 µl 10% D_2_O 1 mM non-deuterated DSS and recombined to make 1.5 ml solutions for NMR analysis.

The extracts were run on a 600 MHz Avance III NMR spectrometer (Bruker) with QCI-P cryoprobe at a calibrated temperature of 298 K to acquire 1D-^1^H spectra. For each sample, an automated programme was set up on the spectrometer using ICON NMR, including measurement of water offset, 90° pulse calibration, locking to D_2_O, tuning and shimming using an excitation sculpting experiment. A 1D-^1^H-NOESY was run with a mixing time of 100 ms, 512 scans and 8 dummy scans, a spectral width of 15.98 p.p.m. (9.59 Hz), 32,768 data points, an acquisition time of 2.27 s and a relaxation delay of 3 s.

The NMR spectra were phased, baseline corrected and had a 1 Hz exponential line broadening window function applied using TOPSPIN 3.6.1 (Bruker) software and then exported into Chenomx 8.4. The water resonance peak between 4.56 and 4.97 p.p.m. was deleted. The spectral peaks were then fit into the Chenomx library of metabolites using the profiler tool to match the peaks to their corresponding metabolites and concentrations.

Metabolites with significantly high abundances and biological importance were divided into four groups: short-chain fatty acids (SCFAs), amino acids, sugars and sugar alcohols and other important metabolites. A time course was plotted to show the change in abundance of each metabolite throughout the antibiotic course.

## Results and discussion

### Composition of gut bacterial communities and *Blastocystis* colonization

Stool samples from days 0, 2, 3, 4, 5, 6, 7, 8, 10, 15, 30 days and 3 months after the start of the antibiotic course were collected, screened for *Blastocystis* presence and processed for 16S rRNA gut microbiome sequencing. *Blastocystis* ST1 was present in samples from D2, D3 and consistently after D8 (Table 1).

**Table 1.** *Blastocystis* colonisation of stool samples collected at different dates of the antibiotic course and 30 days and 3 months after the completion of the course. + indicates the sample is *Blastocystis-*positive and – indicates the sample is *Blastocystis-*negative

Date of antibiotic course	*Blastocystis* +/–
Day 0	−
Day 2	+
Day 3	+
Day 4	−
Day 5	−
Day 6	−
Day 7	−
Day 8	+
Day 10	+
Day 15	+
30 days after the start of the course	+
3 months after the start of the course	+

**Fig. 1.**
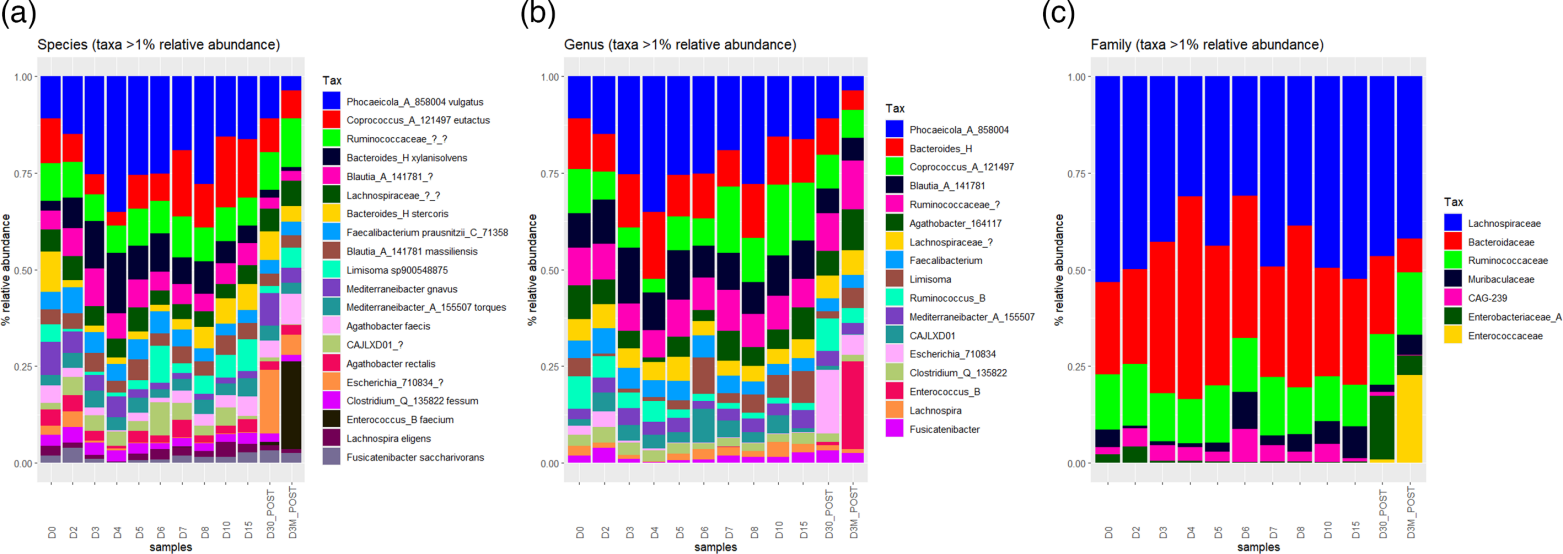
Compositional plots showing the bacterial composition of the gut taxa aggregated to varying taxonomic levels. (a) Species-level. (b) Genus level. (c) Family level. Taxa included made up >1% of total read counts, respectively. Taxa abundances are shown as % relative abundance.

For the microbiome analysis, the most abundant genera (defined here as taxa with a mean abundance exceeding 1% of the total read count across all samples) from all stool samples were plotted as a compositional plot (Fig. 1). A total of 20 species met these criteria. Several genera exhibited patterns of change throughout the antibiotic course, including *Phocaeicola*_A, *Escherichia* and *Enterococcus*_B (Fig. 1). The bacterial communities from samples collected 30 days and 3 months after the start of the antibiotic course were more similar in taxonomic distribution to those taken at D0–D2 and D10–D15 (Fig. 1). The samples collected on D0 and D2 were highly similar to each other. *Bacteroides* and *Phocaeicola* (both from the phylum *Bacteroidota*) were the most abundant genera, becoming more dominant during the first week of the course, with their relative abundances decreasing in the months following completion of the course (Fig. 1). *Phocaeicola* emerged as the dominant genus, showing an increase in relative abundance during the first 4 days of the course, a slight decrease toward the end and a further decline in the months after treatment (Fig. 1). After the antibiotic course, *Phocaeicola* was no longer the dominant genus, with *Escherichia* and *Enterococcus* being the most abundant genera at D30 and D3M, respectively (Fig. 1). *Blastocystis* was not detected on D4, D5, D6 and D7 but was observed again from D8 onwards. The presence of *Blastocystis* coincided with the decrease in abundance of *Bacteroides* and *Phocaeicola* (Fig. 1). The reduced abundance of *Bacteroides* in the presence of *Blastocystis* is a consistent finding across studies globally [8,14, 39,42].

### Impact of antibiotic course and *Blastocystis* colonization on alpha and beta diversity

**Fig. 2.**
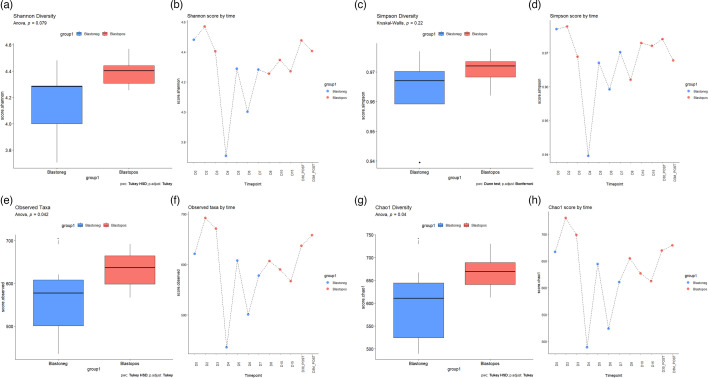
Statistical analysis of diversity scores of samples that were +ve (red) or -ve (blue) for *Blastocystis*. (a,c,e,g) Diversity analysis of samples taken throughout the antibiotics course. (b,d,f,h) Shannon, Simpson, observed (richness), Chao1 scores over time. Kruskal–Wallis H-test and Dunn’s test (Bonferroni *P*-adjust method) or ANOVA and Tukey HSD test were used for statistical analysis (this was based on the normality of the data, determined using the Shapiro test). Kruskal–Wallis/ANOVA scores <0.05, indicating significant differences between the groups.

To measure changes in alpha diversity, we used the Shannon index (which accounts for both evenness and richness), the Chao1 index (a richness index that considers potentially relevant singleton and doubleton ‘rare taxa’), the Simpson index (which measures ‘dominance’ or the extent to which a few taxa constitute most of the reads) and the observed taxa index (representing true richness). These diversity indices were analysed at the antibiotic-negative stage (pooled data from time points D0, D30, D3M) and at the antibiotic-positive stage (pooled data from time points D2, D3, D4, D5, D6, D7, D8, D10 and D15). All four metrics decreased during antibiotic administration. Antibiotics have been associated with acute gut microbiota perturbations marked by a decrease in taxonomic diversity [42,43]. When examined using ANOVA/Kruskal–Wallis tests, the changes among *Blastocystis* +ve/-ve samples were significant for the Chao1 index, with observed taxa indices showing *P*-values of 0.040 and 0.042, respectively, indicating an increase in richness in samples from +ve for *Blastocystis*. Changes between groups for the Shannon and Simpson indices were non-significant (Fig. 2a–d).

The diversity metric scores were also plotted individually over time. The Shannon and Simpson diversity metrics decreased during the antibiotic course; however, the baseline composition recovered following treatment, although a decreasing trend was noted in D3M (Fig. 2a–d). This observation aligns with previous studies in both adults and children whereby core microbiome taxa return to their pre-antibiotics abundance [44]. Chao1 and observed taxa showed sharp reductions at D4, D6 and D15. The reduction in these two indices on D15 contrasts with the increase in the Shannon diversity score, indicating that the recovery in Shannon diversity score towards the end/post-antibiotics course was characterized by a matching reduction in the dominance of the microbiome by a handful of taxa. This could be attributed to the elimination of rare taxa by the antibiotics.

**Fig. 3.**
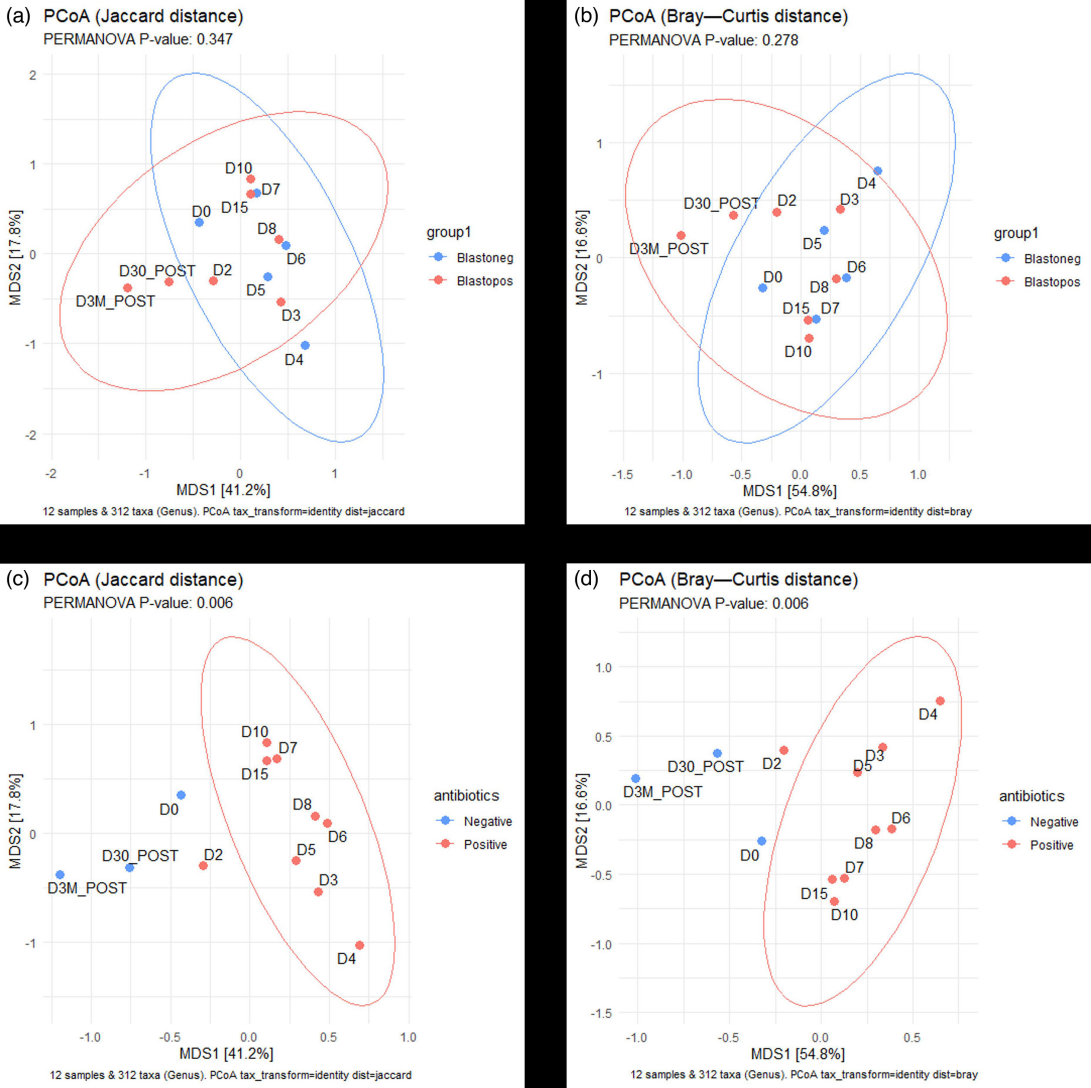
PCoA plot. Plot shows the positions of each sample (based on their microbiome composition) in a dissimilarity matrix (Bray–Curtis distances), different groups are indicated by shape and colour, *Blastocystis*-ve/antibiotic-ve (blue) and *Blastocystis* +ve/antibiotic +ve (red). Statistical analysis of the different groups’ positions was performed using PERMANOVA. The PERMANOVA *P*-value>0.05 indicates that there is no significant difference between the positions of each group’s centroid.

**Fig. 4.**
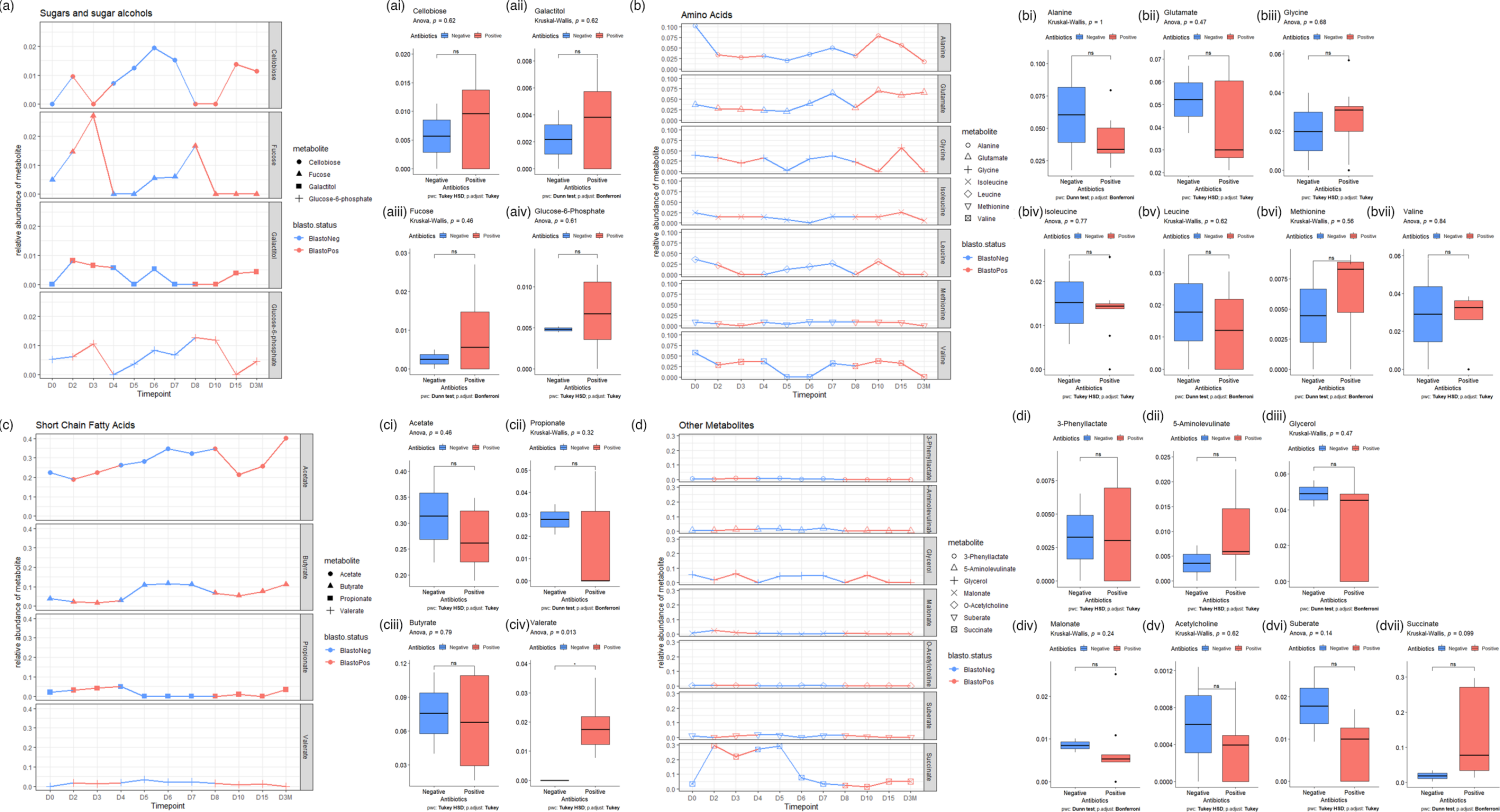
Time course of metabolite abundances of four different groups of metabolites throughout the antibiotic course as well as *Blastocystis* colonization. Colonization status is indicated by the colour *Blastocystis* -ve (blue) and *Blastocystis* +ve (red), and metabolites are indicated by shape. (a) SCFAs. (b) Amino acids. (c) Sugars and sugar alcohols. (d) Other important metabolites.

To compare changes in the microbial composition of the samples, principal coordinate analysis (PCoA) was conducted using the Bray–Curtis dissimilarity matrix and Jaccard distance values (Fig. 3). PERMANOVA was then performed to compare changes in centron positions according to *Blastocystis* colonization status (+ve/-ve) and antibiotic status (+ve/-ve) (Fig. 4). The presence of *Blastocystis* did not have a significant effect on Bray–Curtis or Jaccard distance positions/microbial composition of the samples (Fig. 3a, b). However, the presence of antibiotics significantly impacted the samples’ microbial composition (Fig. 3c, d). Both the Bray–Curtis and Jaccard distance values showed significantly distant centroids between the antibiotic +ve and -ve groups.

### Biomarker analysis of stool samples for *Blastocystis* colonization

Having shown that increases in diversity metrics occur with the presence of *Blastocystis* in stool samples, LEfSe was used to look for biomarkers of this change (Fig. S2, available in the online Supplementary Material). LEfSE [37] identifies taxa whose presence/absence allows for the best discrimination of a sample into one of the two groups, in this case Blasto +ve or Blasto -ve [37]. LEfSE employs Kruskal–Wallis tests to determine significant taxa. The effect size of these significant taxa is then plotted. An LDA score (effect size) >2 or <–2 is considered a strong effect. Three taxa were significant discriminators for *Blastocystis-*negative samples, namely, an unclassified member of the family *Marinifilaceae*, *Bacteroides thetaiotaomicron* and *Dialister invisus*. Sixteen taxa were considered indicative of the *Blastocystis-*positive group. These taxa consisted of: *Alistipes putridinsis*, *Dysosmobacter welbionis*, *Mediterraneibacter faecis*, *CAG-177* (member of the family *Actualibacteraceae*), *CAG-273* (member of the class *Clostridia*), *PeH17* (of the order *Christensenellales*) and *SFLA01* (of the order *Oscillospirales*), as well as unclassified members from the genera *Collinsella*, *Anaerotruncus*, *Butyricimonas*, *Angelakisella*, *Onthomonas*, *SFEL01* (of the order *Christensenellales*) and *DTU012* (of the class *Limnochordia*). There was also an unclassified member of the order *Christensenellales* and the class *Coriobacteriia. Blastocystis* has been associated with gut-healthy individuals [13]. The most significant bio-marker taxa for *Blastocystis*-positive samples include *Clostridium*, a common human gut taxon associated with homeostasis (clusters XIVa/IV) [35], and *Alistipes*, a genus described in 2003 and thought to contain species protective against gut inflammation such as colitis [45]. It is intriguing to hypothesize that the presence of *Blastocystis* and the increased abundance of *Bifidobacterium longum* could indicate that the patient was on their way to recovery. Hence, we urge that future microbiome studies focusing on dysbiotic gut should also include *Blastocystis*.

### Metabolite composition during the antibiotic course

The metabolite extracts from the stool samples were subjected to 1D ^1^H NMR. Based on their chemical properties, four groups of metabolites were detected, including SCFAs (Fig. 4a), amino acids (Fig. 4b), sugars and sugar alcohols (Fig. 4c) and other relevant metabolites (Fig. 4d). Previous metabolome investigations at a single timepoint on *Blastocystis-*positive and -negative individuals showed a decreased abundance of certain metabolites in the former [2]. Specifically, alanine, glycine, histidine, isoleucine, methionine, threonine, tryptophan and valine all decreased, suggesting an anti-inflammatory role of *Blastocystis*. In support of this, significant increases in certain amino acids in the stool of IBD patients have been found [46]. Herein, in a time course metabolome of a single individual, all the amino acids, particularly alanine and valine, showed a decrease mid-course and recovery towards the end of the course. Moreover, all amino acids but glutamate showed a large decrease post-course (Fig. 4b).

Regarding SCFAs, the abundance of acetate increased throughout the first week of the treatment but decreased during the second week and then recovered post-course. Butyrate increased after the first 4 days, decreased on D7 and recovered post-course. Whether these alterations reflect changes in absorption or loss remains an open question. Cellobiose was the most impacted sugar and showed a large increase during the first week and then a decline and recovery during the second week (Fig. 4c). Malonate steadily declined for the first 5 days and was undetectable by D6. It then recovered on D7, D8 and D10 but became undetectable on D15 and post-course (Fig. 4d). Succinate sharply increased from D1 to D2 and stayed high until D5, when it declined again. O-Acetylcholine declined for the first 5 days then recovered on D6 and D7 but disappeared during the second week and did not recover post-course. Notably, acetylcholine in the gut plays a role in intestinal homeostasis; hence, its disruption could potentially aggravate inflammation [47].

**Fig. 5.**
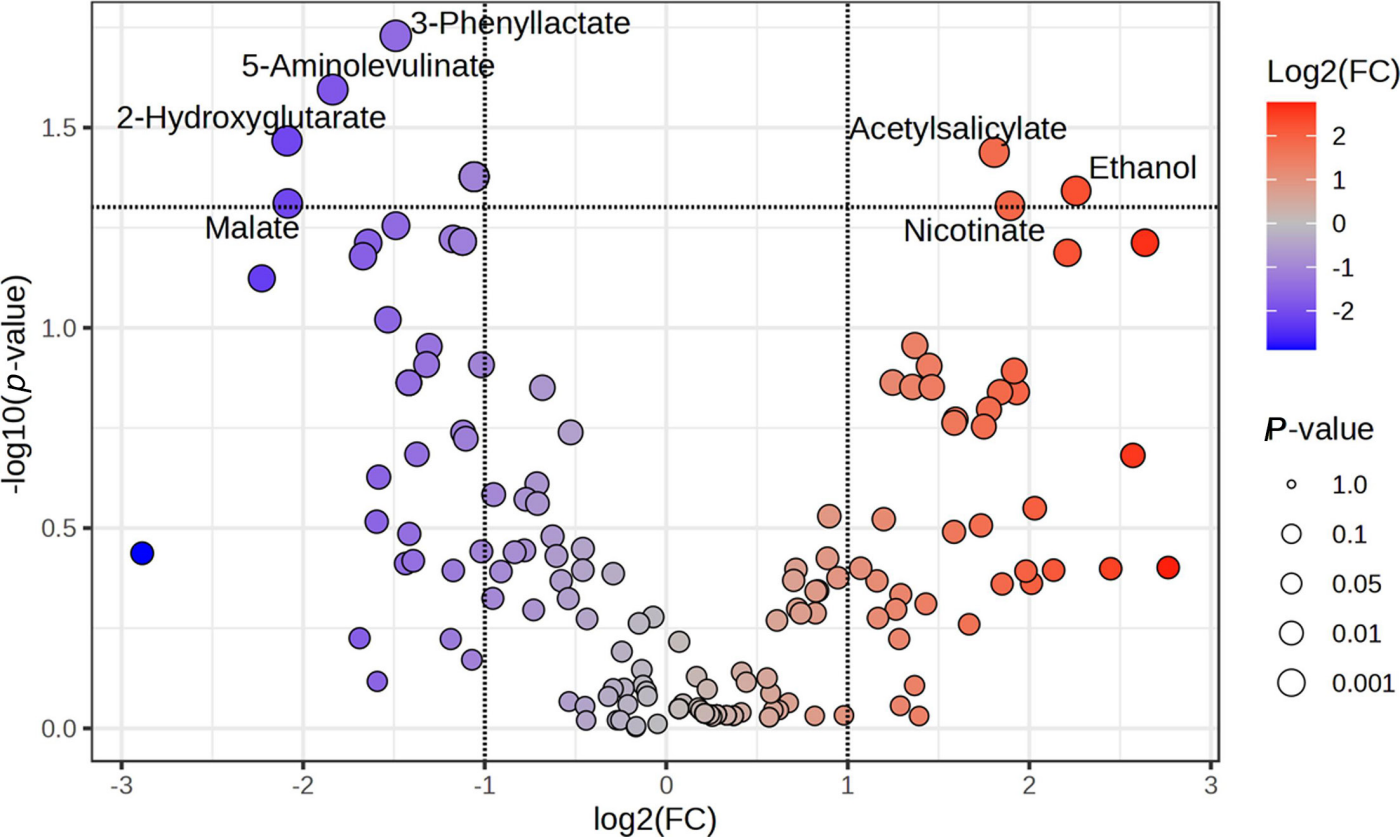
Volcano plot showing the log-fold change of metabolites within the samples; metabolites are coloured according to their increased presence (blue) or decreased presence (red) in *Blastocystis*-positive samples. Named/annotated metabolites are those with a significant *P*-value of <0.05.

Having observed the changes of the most abundant metabolites in the four groups (in antibiotic +ve/-ve samples), log-fold change analysis (Fig. 5) was performed to find metabolites undergoing significant changes in accordance with *Blastocystis* colonization status. Results were depicted as a volcano plot (Fig. 5). Metabolites with a significant *P*-value (<0.05) are named/annotated. Metabolites were coloured according to the direction of their change during *Blastocystis* colonization (blue, reduction; red, increase). The volcano plot showed four metabolites undergoing significant reduction in the presence of *Blastocystis* (malate, 2-hydroxyglutarate, 5-aminolevulinate and 3-phenyllactate) and three metabolites exhibiting a significant increase in the presence of *Blastocystis* (ethanol, acetylsalicylate and nicotinate).

### Impact of microbial changes and *Blastocystis* colonization on metabolite composition during the antibiotic course

**Fig. 6.**
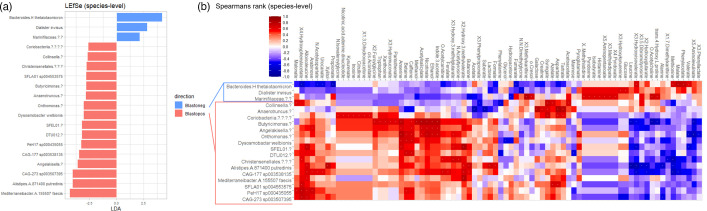
Linear discriminant analysis (LDA) effect size (LEfSe) plot at species-level, shown with correlated metabolites. Comparisons are undertaken with significant LEfSe taxa for *Blastocystis* status (+ve/-ve). LEfSe plot LDA scores indicate the presence/increased abundance of each taxon to discriminate between two conditions, *Blastocystis* +ve (red) and *Blastocystis* -ve (blue). Taxa with LDA scores between −2 and 2 are considered insignificant ‘biomarkers’ and are not included in the plot (B/D). Taxa shown to be statistically significant linear discriminants of microbiome data were correlated with the metabolomics results. Shown are Spearman’s rank correlations; white stars indicate a significant *P*-value for the correlation (however, Benjamini-Hochberg *P*-adjust revealed that none of these were significant).

Three taxa, namely *Bacteroide*s *thetaiotaomicron*, *Dialister invisus* and the family *Marinifilaceae,* significantly increased in *Blastocystis* -ve samples (Fig. 6a). The increase in the abundance of *Bacteroides thetaiotaomicron* and *Dialister invisus* correlated negatively with the abundance of the SCFA propionate (Fig. 6b). Past studies have shown that propionate promotes satiety and reduces cholesterol [48,49], suggesting that the increase in the abundance of these taxa may have a negative effect on GI health. In contrast, the amino acids alanine and leucine correlated positively with an increase in the abundances of *Bacteroides thetaiotaomicron* and *Dialister invisus* (Fig. 6b), aligning with previous studies [1]. The species SFLA01 sp004553575 (a member of the order *Oscillospirales*) and PeH17 sp000435055 (a member of the order *Christensenellales*), as well as members of the genera CAG-273 (a member of the class *Clostridia*) and CAG-177 (of the family *Acutalibacteraceae*), should be noted that the 16S gene cannot be used to identify species within the genera CAG-273 and 177.

Increased levels of *Blastocystis* +ve samples (Fig. 6a) correlated with an increase in sugars (e.g. arabinose) and sugar alcohols (e.g. xylitol) (Fig. 6b). Arabinose is poorly absorbed by the gut [49]; instead, it is metabolized by gut microbiota as a carbon source. Various benefits, such as the reduction of obesity and amelioration of colitis, have been attributed to arabinose. However, further research is needed to disentangle its role in the gut and its connection with the presence of *Blastocystis*. Xylitol, a natural sweetener, enhances the synthesis of propionate in the colon through the cross-feeding of gut microbiota [50]. Its increase and decrease correlate with the levels of propionate, suggesting that xylitol might be an important component in the probiotic health benefits of propionate. Therefore, the interplay between potential host consumption of xylitol, certain bacterial taxa and propionate in *Blastocystis* +ve suggests a potential health benefit.

## Conclusions

This case study pilots an approach that integrates antibiotic treatment in an IBS patient, examines the presence of *Blastocystis* and analyses the overall gut microbiome and metabolome. The gut microbial composition was significantly altered by a 14-day course of a three-antibiotic cocktail, with a notable decline in microbial diversity mid-course. This longitudinal study revealed that *Blastocystis* becomes undetectable at certain time points using the common diagnostic method (qPCR). This raises several questions: does the abundance of *Blastocystis* reach a threshold that is not detectable by qPCR? If so, what factors determine this reduction? Are the changes in *Blastocystis* abundance related to changes in the microbiome and/or metabolome of the host? Given these questions, future research should focus on longitudinal studies in larger cohorts. This will provide a more comprehensive understanding of *Blastocystis* as a stable resident of the gut.

## Supplementary material

10.1099/acmi.0.000926.v4 Uncited Supplementary Material 1.

## Abbreviations

blast: basic local alignment search tool
DADA2: Divisive Amplicon Denoising Algorithm 2
GG2: GreenGenes2
GI: gastrointestinal
IBS: irritable bowel syndrome
LEfSe: linear discriminant effect size
PCoA: principal coordinate analysis
qPCR: quantitative PCR
RT: room temperature
SCFAs: short-chain fatty acids
*SSU* rRNA: small subunit rRNA
