# Supplementary material for: Effect of antibiotic administration on Blastocystis persistence and gut microbiome–metabolome dynamics in an irritable bowel syndrome longitudinal case study
Source: Access Microbiol. 2025 Jun 26;7(6):000926.v4. doi: 10.1099/acmi.0.000926.v4 (PMC12202796; doi:10.1099/acmi.0.000926.v4)
Supplement: Uncited Supplementary Material 1. [file acmi-7-00926-s001.pdf]

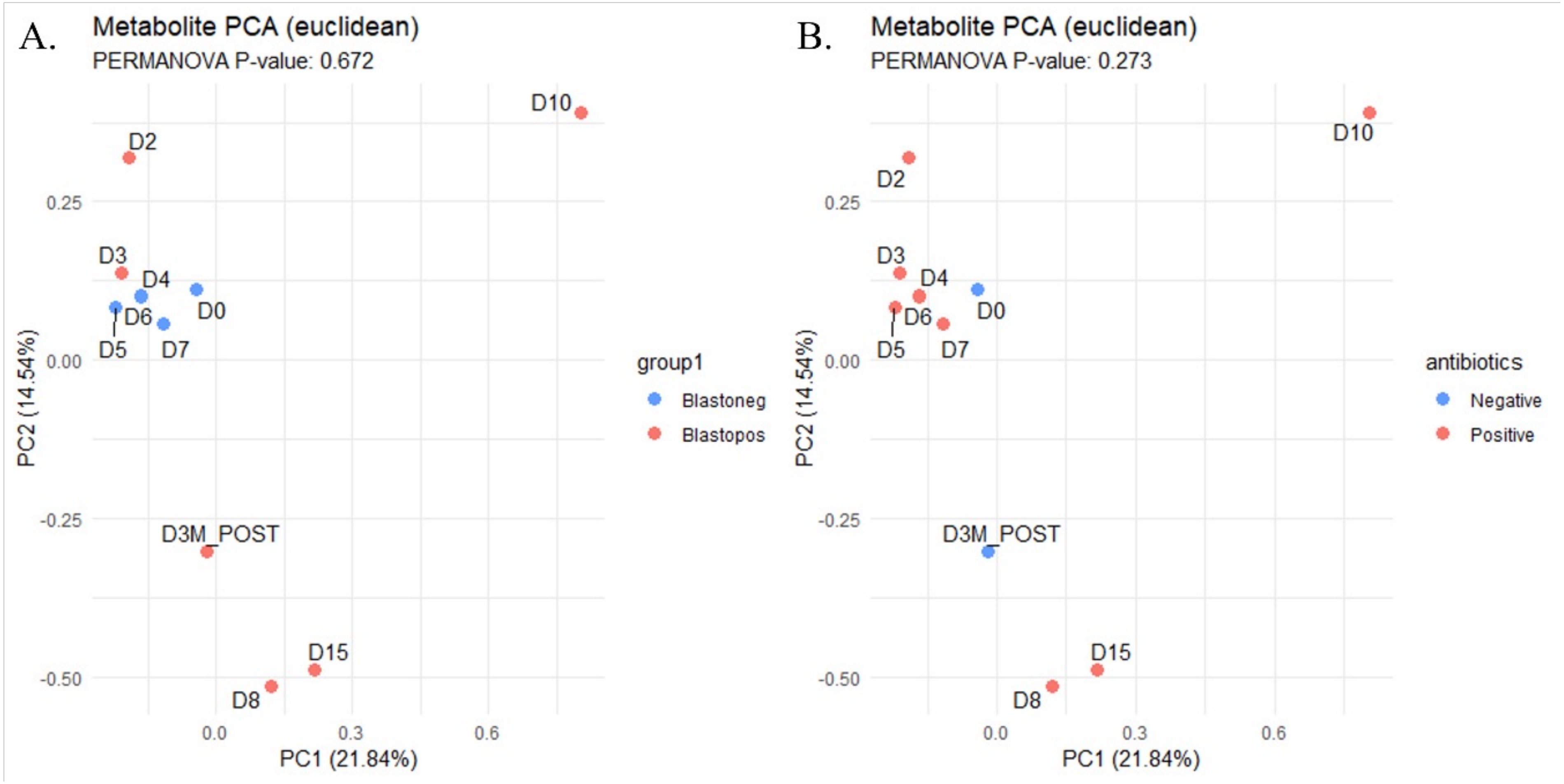

Supplementary Figure 1: Principle component analysis (PCoA) plot. The plot shows the positions of each sample (Euclidean distances) based on each sample's metabolite composition. Different groups (*Blastocystis* status and antibiotics) are indicated by colour. Statistical analysis of the distinctive groups' positions was performed using PERMANOVA. The PERMANOVA P-value >0.05 indicates no significant difference between the positions of each group's centroid.

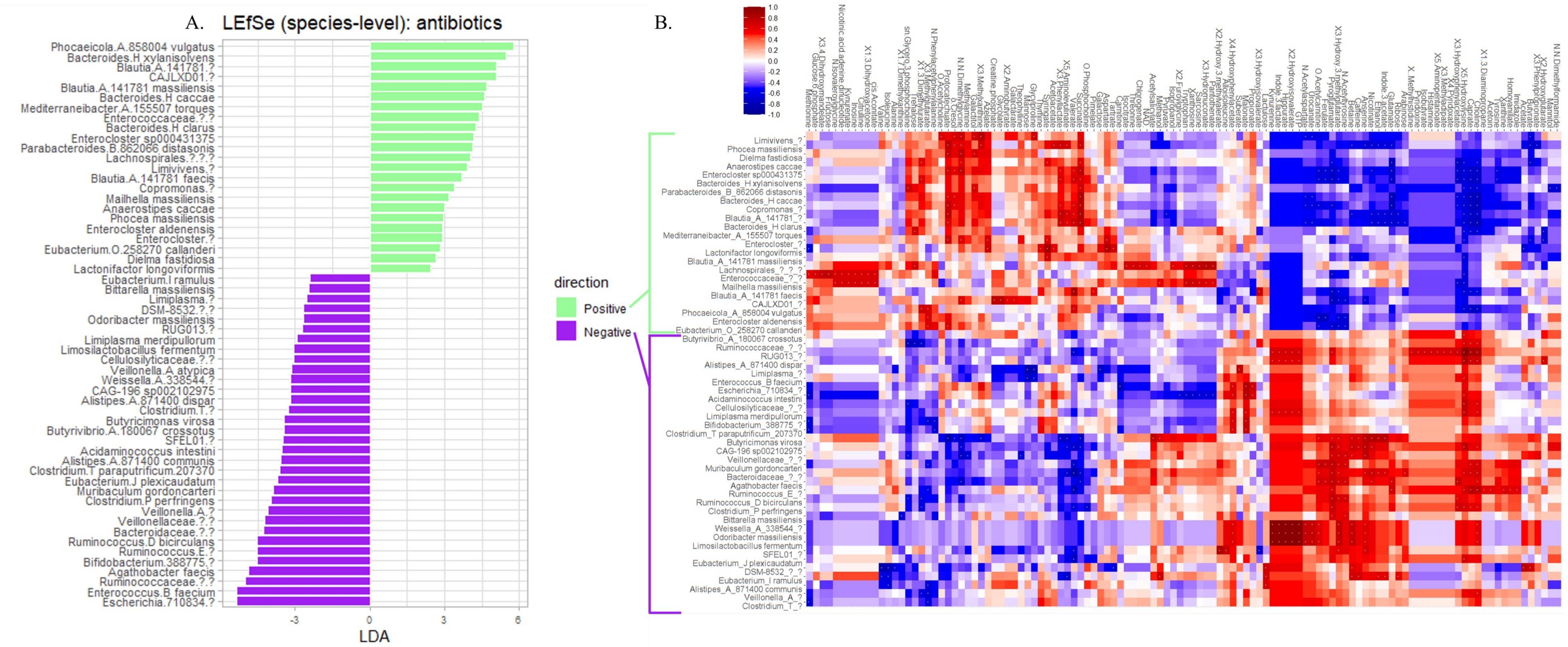

Supplementary Figure 2. Linear Discriminant Analysis (LDA) Effect Size (LEfSe) plot at Species-level, shown with correlated metabolites. Comparisons are undertaken with significant LEfSe taxa for antibiotics status (+ve/-ve). A/C). LEfSe plot LDA scores indicate the presence/increased abundance of each taxon to discriminate between two conditions, Antibiotics +ve (green) and Antibiotics -ve (purple). Taxa with LDA scores between -2 and 2 are considered insignificant 'biomarkers' and are not included in the plot. B/D). Taxa shown to be statistically significant linear discriminants of microbiome data were correlated with the metabolomics results, shown are the Spearman's rank correlations, white stars indicate a significant P-value for the correlation (however, Benjamini-Hochberg P-adjust revealed none of these was significant).
